# Vertebral osteomyelitis concurrent with emphysematous pyelonephritis and psoas abscess

**DOI:** 10.1002/ccr3.3447

**Published:** 2020-10-28

**Authors:** Kojiro Tanaka, Naoki Yonezawa, Tetsuhiro Takei

**Affiliations:** ^1^ Department of Emergency and Critical Care Medicine Yokohama City Minato Red Cross Hospital Yokohama Japan

**Keywords:** *Escherichia coli*, osteomyelitis, psoas abscess, pyelonephritis, surgery

## Abstract

Vertebral osteomyelitis associated with concurrent emphysematous pyelonephritis and psoas abscess is rare. Coexistence of these entities is a potentially life‐threatening condition, necessitating aggressive intervention.

## Graphical abstract

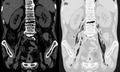

Vertebral osteomyelitis associated with concurrent emphysematous pyelonephritis and psoas abscess is rare. Here, vertebral osteomyelitis developing secondary to hematogenous seeding of *Escherichia coli* from a primary urinary tract infection was later complicated by contiguous spread to the psoas. Coexistence of these entities is a potentially life‐threatening condition.

A 69‐year‐old man with uncontrolled diabetes and prostatic hyperplasia presented with a 7‐day history of lower back pain. He was afebrile and hemodynamically stable, his leukocyte count was 15.0 × 10^3^/µL, C‐reactive protein concentration was 29.6 mg/dL, and the glycated hemoglobin level was 10.9%. Abdominopelvic computed tomography revealed gas widely distributed across the left renal parenchyma, bilateral psoas, and third lumbar vertebra (Figure 1). Lumbar magnetic resonance imaging revealed vertebral osteomyelitis (Figure 2). Despite initial intravenous meropenem and vancomycin and then cefazolin (2 g every 8 hours), the psoas abscess had to be drained percutaneously on day 10 because of remittent fever for 5 days. However, septic shock developed, which necessitated emergency surgical debridement for vertebral osteomyelitis on day 11. Debridement, drainage, urine, and blood cultures revealed fully susceptible *E coli*. After a 12‐week intravenous antibiotic therapy, the patient gradually improved and is currently undergoing rehabilitation.

**FIGURE 1:**
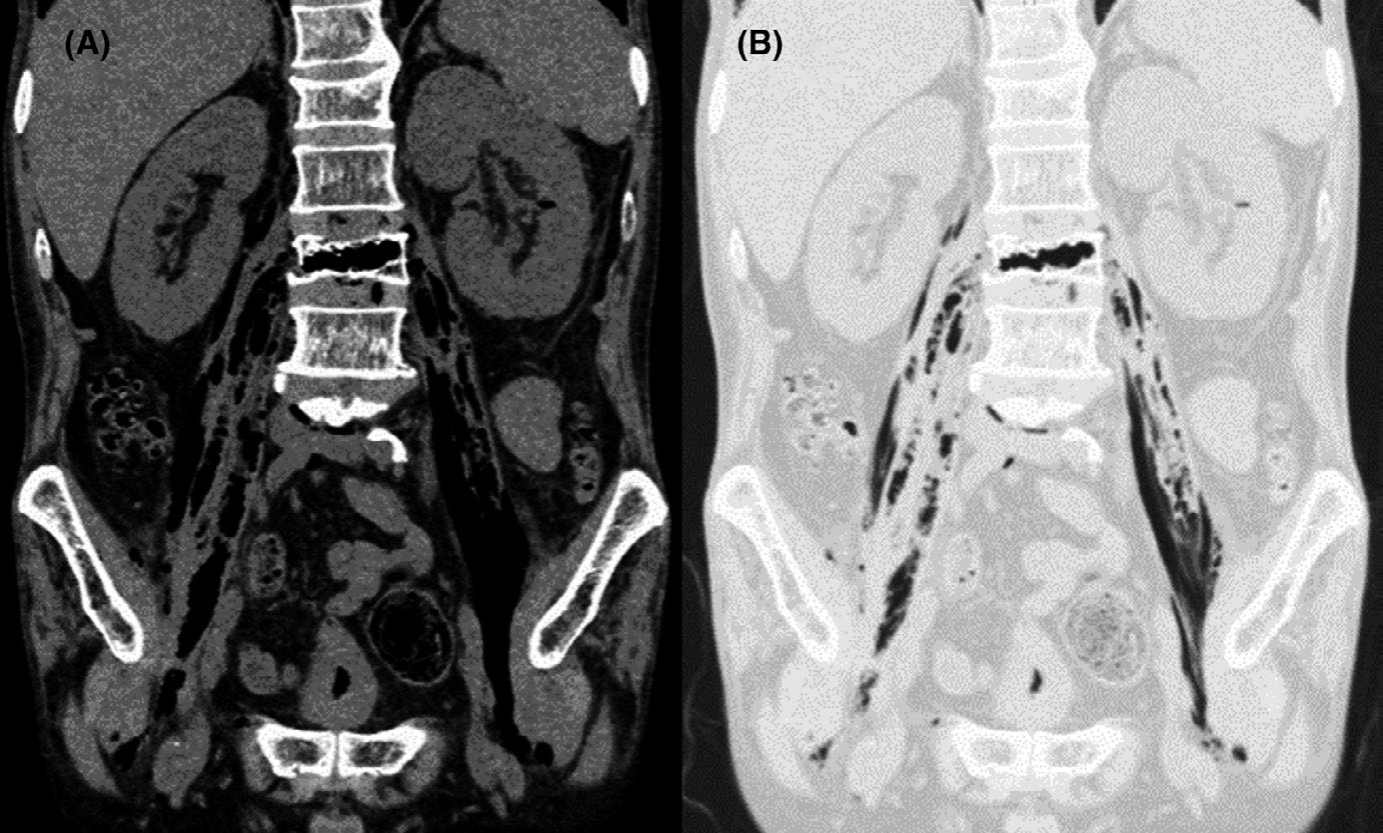
Coronal view on abdominopelvic computed tomography on admission, showing gas widely distributed across the left renal parenchyma, bilateral iliopsoas, and third lumbar vertebra. (A) Standard window setting. (B) Pulmonary window setting

**FIGURE 2:**
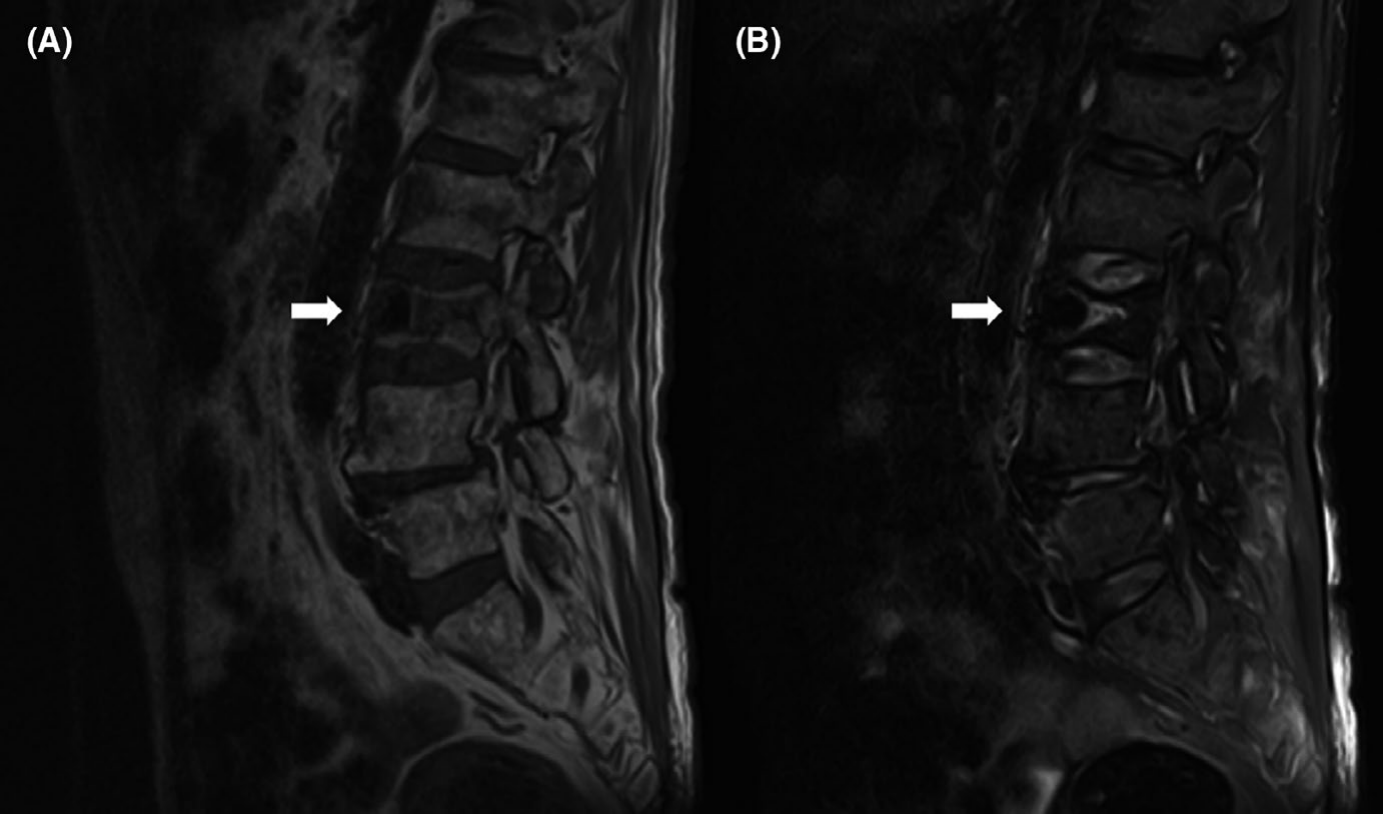
Sagittal magnetic resonance imaging of the lumbar spine, revealing vertebral osteomyelitis of the third lumbar vertebra and a compression fracture, as evidenced by the low‐intensity region on the T1‐weighted image (Panel A, arrow) and the high‐intensity region on the fat‐suppressed T2‐weighted image (Panel B, arrow)

Vertebral osteomyelitis probably resulted from the hematogenous spread of urinary tract infection to the vulnerable vertebral body and contiguous spread to the psoas; it is potentially life‐threatening, with high morbidity and mortality rates.^1^ The coexistence of lumbar vertebral osteomyelitis, bilateral psoas abscess, and left emphysematous pyelonephritis is rare,complicated vertebral osteomyelitis necessitates aggressive intervention.

## CONFLICTS OF INTEREST

The authors declare that they have no conflict of interests.

## AUTHOR CONTRIBUTIONS

KT: cared for the patient, and initiated and wrote the manuscript. NY: collected, analyzed, and interpreted the clinical data, and revised the manuscript. TT: interpreted the clinical data and critically reviewed the manuscript. All authors of this paper have read and approved the final version submitted.

## ETHICAL APPROVAL

The study was published with written consent of the patient.
